# Biocompatibility and Osseointegration of the Biomimetically Coated and Water-Soluble Eggshell Membrane Protein Cross-Linked Ti Alloy Screws

**DOI:** 10.61186/ibj.3939

**Published:** 2023-10-24

**Authors:** Mehmet Bahadır İlik, Ercan Durmuş, İlhami Çelik

**Affiliations:** 1 Sağlık Bakanlığı, Alanya ADSM, Oba Mahallesi, Fidanlık Caddesi, No:85, Alanya, Antalya, Türkiye;; 2 Selçuk University Faculty of Dentistry, Department of Oral and Maxillofacial Surgery, Kampüs 42080, Konya, Türkiye;; 3 Selçuk University Faculty of Veterinary Medicine, Department of Histology and Embryology, Kampüs 42031, Konya, Türkiye

## Abstract

**Background:**

The surface properties of dental and orthopedic implants are directly related to their osseointegration rate. Coating and/or modifying the implant surface might reduce the time of healing. In this study, we aimed to examine the effects of a hybrid surface consisting of a brushite surface coating and cross-linked water-soluble eggshell membrane protein on the osseointegration of Ti screws under in vivo conditions.

**Methods:**

Twenty Ti alloy screws were implanted monocortically in anteromedial regions of New Zealand rabbit tibiae. Ten screws were untreated and used as controls. The remaining 10 screws were coated with calcium phosphate and following cross-linked with ostrich eggshell membrane protein. All rabbits were sacrificed six weeks after the surgery. Peri-screw tissues were evaluated by µ-CT, histological and histomorphometrical methods.

**Results:**

The μ-CT assessments indicated that the experimental group had significantly higher mean BSA and TbN than those of the control group (*p *˂ 0.05). BV, TbSp, TbTh, and BMD scores of the control and experimental groups were quite similar (*p *> 0.05). The vascularization score of the experimental group was significantly higher than the control group (4.29 vs. 0.92%). No sign of the graft-versus-host reaction was observed.

**Conclusion:**

Our findings reveal that coating Ti alloy implants with calcium phosphate cross-linked with ostrich eggshell membrane protein increases the osseointegration of Ti alloy screws by increasing the bone surface area, number of trabeculae and vascularization in the implant site.

## INTRODUCTION

Biomaterials are synthetic, natural or modified materials used in repairing the organ or body function and interact with biological systems for a medical purpose^[^^1^^]^. Depending on the chemical nature, biomaterials can be classified as metals, polymers, and ceramics. Calcium phosphate-based ceramics have a similar composition to bone tissue and can be used in the reconstruction of the skeletal system of the body^[^^2^^]^. this kind of biomaterials possess bioactivity, osteoconductivity, and surface properties that allow them to bind to the bone morphogenic proteins^[^^3^^]^.

The biomimetic coating technique is a method in which hydroxyl apatite and octacalcium phosphate crystals are deposited onto the substrate material, placed in a supersaturated calcium phosphate solution. In this method, compared to the plasma deposition technique, the reactions occur at significantly lower temperatures. By biomimetic coating method, osseointegration and ossteoconductivity of Ti alloy implants can prominently be improved^[^^4^^]^. In addition, this method allows for the cross-linking of various bioactive proteins with inorganic materials, as a surface layer^[^^5^^]^. The organic matrix of the natural bone tissue is formed by the three-dimensional organization of collagen molecules in the extracellular matrix, which is mainly composed of collagen fibers^[^^6^^]^. Collagens are basically composed of type I and a lesser amount of type V collagen. During biological mineralization of the osseous tissues of mammals, collagen fibers in the extracellular matrix are involved in bone formation together with calcium phosphate crystals, which are precipitated on the collagen fibers^[^^7^^]^.

Eggshell membranes are located under the calcified eggshell as two definite layers^[^^8^^]^ and contain type I, type V, and type X collagens^[^^9^^-^^11^^]^. When eggshell membranes are used as a biomaterial, physical changes cannot be applied on these membranes due to disulfide bonds in their molecular structure. Water-soluble eggshell membrane proteins, compared to native eggshell membranes, could are convenient materials for using as a biomaterial^[^^12^^]^. Therefore, various methods have been developed to obtain water-soluble proteins from eggshell membranes^[^^13^^]^. The water-soluble eggshell membrane proteins contain the same amino acids as the eggshell membrane. However, owing to the high surface energy and the more hydrophilic surface, the water-soluble eggshell membrane proteins provide a favorable environment for the adhesion and proliferation of more fibroblasts^[^^12^^]^. Additionally, the resulting product promotes the reconstruction of collagen matrix and reduces the solubility of the collagen matrix in different solvents^[^^13^^,^^14^^]^. Collagen improves the physical properties of the matrix by increasing its elasticity, denaturation temperature, and fibroblast migration to the material surface. Hence, water-soluble eggshell membrane proteins are promising products that can be used in tissue engineering applications^[^^15^^,^^16^^]^.

In the present study, the biocompatibility and osseointegration of Ti alloy screws coated with calcium phosphate and cross-linked with water-soluble ostrich eggshell membrane protein were evaluated by µ-CT, histological and histomorphometric methods.

## MATERIALS AND METHODS

**Study groups**

A total of 20 Ti alloy screws (grade 5 [Ti6Al4V]; 1.95 mm diameter and 6 mm length) were employed in this study. Ten unprocessed screws (LeFórte, Jeil Medical, South Korea) were used as the control group. The experimental group included 10 calcium phosphate-coated, cross-linked screws with water-soluble ostrich eggshell membrane protein. 

**Animals and surgical procedures**

Five adult male New Zealand rabbits (*Oryctolagus cuniculus*) were selected for the study. Prior to the initiation of experiments, the animals were prepared for surgery during a one-week habituation period in the Experimental Medicine Research and Application Center of Selçuk University, Konya, Türkiye. Two screws, one unprocessed and the other processed, were implanted in the anteromedial region of the tibia of the animals under general anesthesia, induced by the intramuscular injection of ketamine hydrochloride (35 mg/kg; Ketanes, Alke, İstanbul, Türkiye) and xylazine (5 mg/kg; Rompun, Bayer, Leverkusen, Germany). Animals were sacrificed by intraperitoneal overdose (100 mg/kg) injection of pentobarbital sodium (Nembutal; 100 mg/ml; Abbott Laboratories, Chicago, IL, USA) at the 6^th^ week postoperatively. Tibia samples containing the screws were dissected and placed in phosphate-buffered saline (0.1 M; pH 7.4).

**Coating and protein adsorption to the surface of screws**

Surface of the screws were activated and coated with 10× simulated body fluid as previously described^[^^17^^]^. Briefly, coating stock solution was prepared from the same body fluid with final ion concentration of 1137.5 mM and pH 6.5. For coating, the surface of screws was placed in the coating solution with continuous stirring and kept at room temperature (22 ºC) for 6 h. The coating solution (270 ml) was renewed every 2 h for 6 h.

**Characterization of the surface coating**

The morphology of the surface coated screws was assessed by SEM (Zeiss EVO LS10, Carl Zeiss® NTS, Oberkochen, Germany), and the thickness of the calcium phosphate layer was measured. The chemical composition of the coating was determined by energy-dispersive X-ray analysis. Assessment of the surface of coating was performed by utilizing an X-ray diffraction analyzer (Bruker D8 advance, Kontich, Belgium). The surface of the sample was scanned at 1.54060Å, with a scan speed of 2°/min between 10-50 degrees. The acquired X-ray diffractogram was evaluated by International Center for Diffraction Data using the DIFFRAC.EVA software (Bruker®).

**Cross-linking of soluble eggshell membrane protein to the surface coated screws**

The soluble eggshell membrane protein was prepared according to previous investigations^[^^13^^-^^18^^]^. Briefly, 3 g of the powdered eggshell membrane was treated with 300 ml of performic acid at 25 °C for 24 hours, and the solution was filtered through a glass filter. The resulting filtrate was washed with distilled water, and then 2 g of the obtained intermediate product was added to 20 mg of pepsin (Sigma-Aldrich, St. Louis, USA) solution (3200 IU/mg) in acetic acid (0.5%). The product, while continuously stirring, was incubated at 25 °C for 48 hours. At the end of the incubation period, enzymatic activity was stopped by the addition of pepstatin (Sigma-Aldrich) at a concentration of 0.2 mg/100 ml, and then the solution was centrifuged. The supernatant, which was rich in membrane proteins, was separated from the solution by dialysis. After powdering through lyophilization, the soluble eggshell membrane protein was cross-linked to the surface coated screws as described previously^[19]^. For this purpose, a protein solution was prepared by dissolving 50 μg/ml (w/v) of soluble eggshell membrane protein in 2% acetic acid. The pH of the solution was adjusted to 7.5 by 0.1 M of sodium bicarbonate to prevent dissolving the crystals. The coated screws were dipped into the protein solution under 400 mbar vacuum at 40 °C and kept for 4 h. At the end of the dipping period, the screws were dried in porcelain pots at 30 °C and kept in 8% (v/v) glutaraldehyde solution for 3 h, in order to cross-linking with the water-soluble eggshell membrane proteins. The screws were then washed with 3% sodium bisulfite by shaking in distilled water for 24 hours, and the washing solution was refreshed every 6 h. The screws were dried at 40 °C, sterilized with ethylene oxide and stored in the refrigerator until use. 

**Micro-computed tomography**

The tibia samples containing the grade 5 Ti alloy screws were scanned with a µ-CT scanner (SkyScan CT Analyser; Bruker®, Kontich, Belgium) at 100 kV, 100 µA, and medium resolution of 12 µm by using an aluminum filter. Digital images were recorded using a high-resolution camera (Hamamatsu C9,300® 11 Mp; Kontich). The quality of bone tissue surrounding the body and tip of each screw (1 mm in width and 3 mm in length) was evaluated using the µ-CT analysis program (SkyScan 1172®, version 1.5 Software, Aartselaar, Belgium). Total BV, BSA, TbN, TbSp, TbTh, and BMD in the defect area were measured. 

**Histology and histomorphometry**

Following micro µ-CT analysis, the tibia samples were immediately fixed in 10% buffered formalin for 72 hours and decalcified in 10% EDTA (Titriplex; Merck, Darmstadt, Germany) at 4 °C for 3 months. After decalcification, the samples were embedded in paraffin blocks by routine histological procedures and then sectioned. The sections were stained with hematoxylin-eosin, toluidine blue, Crossmon’s trichrome, and Pappenheim's panoptic stains^[^^17^^]^. For osteoclastic cell staining, acid phosphatase was demonstrated histochemically. Specimens were examined under a light microscope (Nikon Eclipse E 400 equipped with DS Camera Head DS-5M; Nikon Corporation, Chiyoda-ku, Japan). Histomorphometric analyses were performed on the recorded digital images by using image processing software (BS 200 PRO, BAB Soft®, Ankara, Türkiye). The mean percentages of newly formed bone, osteoid and connective tissues, blood vessels, and osteoclast cells around the screws were determined in one field of microscope area. 

**Statistical analysis**

The data obtained by histomorphometric and µ-CT measurements were analyzed by SPSS (version 15) software. The significance of the differences between the two study groups was determined using the Mann Whitney-U test. The *p* < 0.05 values were considered as statistically significant. 

## RESULTS

**Characteristics of the coating surface**

**Fig. 1:**
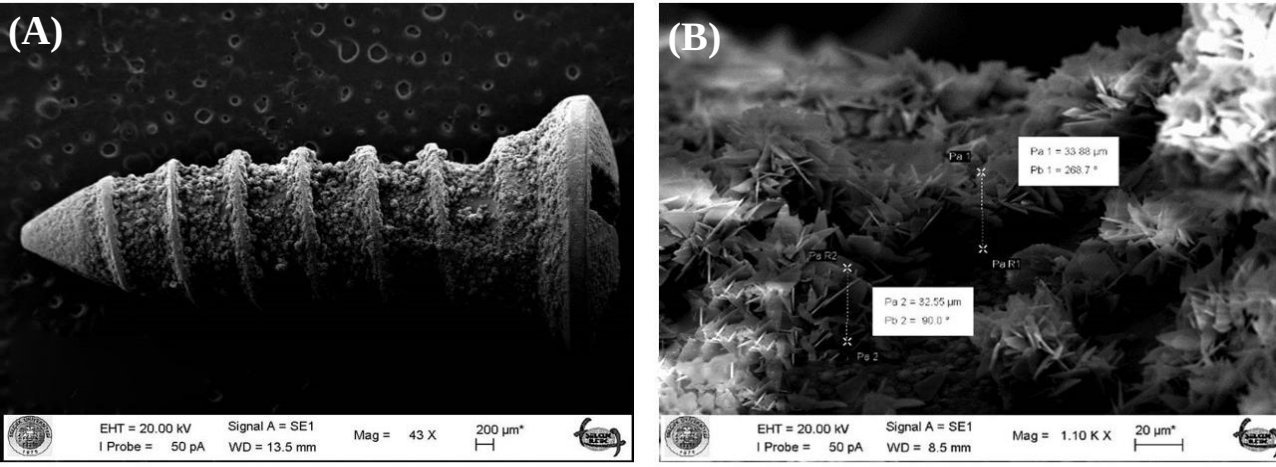
SEM image of the surface layer of a coated screw. (A) Calcium phosphate layer (magnification bar: 200 µM) and (B) the thickness of the layer in two different regions (magnification bar: 20 μm)

**Fig. 2:**
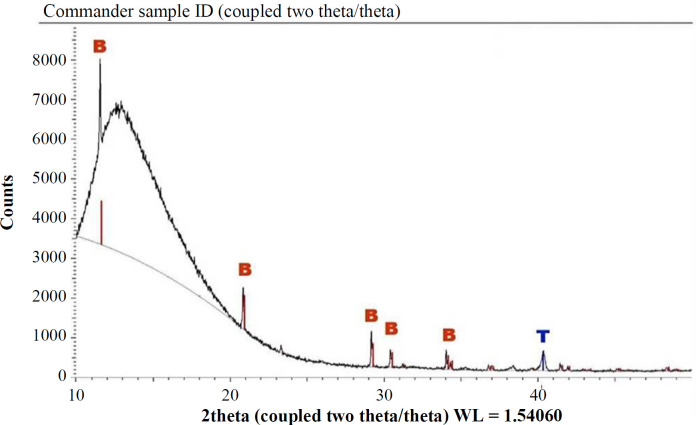
X-ray diffraction analysis of the calcium phosphate-coated surface. Brushite peaks (B) and Ti alloy (T) are highlighted in red and blue peaks, respectively

A sufficient calcium phosphate layer was precipitated on the screws by biomimetic coating (Fig. 1A). The coating was non-homogeneous and its thickness varied between 32-48 µm (Fig. 1B). Energy dispersive X-ray scores of the coating surface showed the mean Ca/P molar ratio of 0.95 ± 0.03. X-ray diffraction scores of the samples exhibited a set of reflection peaks resembling brushite (CaHPO_4_•2H_2_O, dicalcium phosphate dihydrate, Inorganic Crystal Structure Database, code: 16,132) crystals (Fig. 2).

**µ-CT analysis results**

**Fig. 3:**
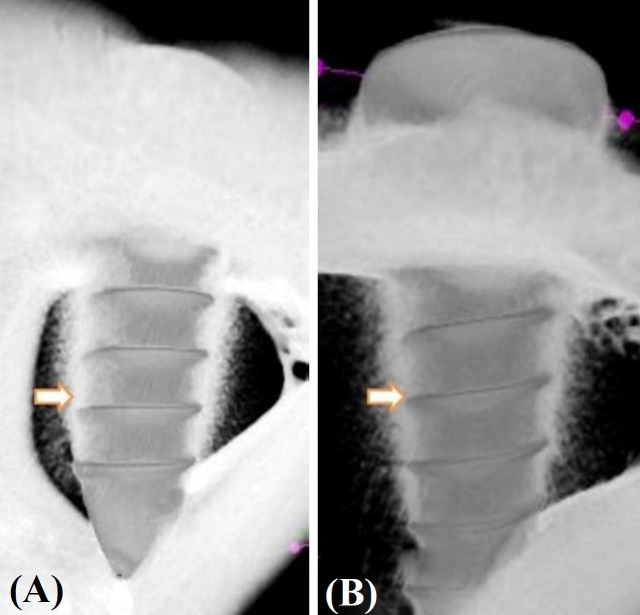
µ-CT images of the (A) control and (B) experimental Ti screws. Arrows show the newly formed bone tissue

Results of µ-CT analysis showed that the mean BSA and TbN values of the experimental groups were significantly (*p* ˂ 0.05) higher than those of the control group. However, there were no significant differences (*p* > 0.05) among the TbTh, BV, BMD, TbSp, and TbTh scores in the study groups (Fig. 3A and 3B; Table 1).

**Histologic and histomorphometric analysis**

**Fig. 4:**
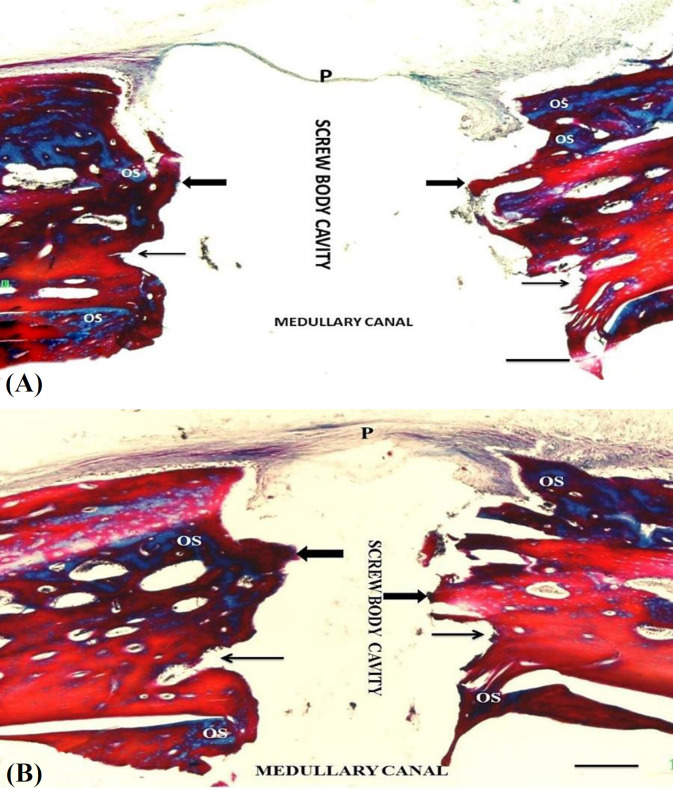
A section from the screw-implanted site in the (A) control and (B) experimental groups. A thin periosteum (P) continuously covers the implantation site. (A) Intercortical bone surfaces adjacent to the screw are covered with thin newly formed primary bone tissue (thick arrows). Osteoid tissue (OS) regions are seen in the deep regions of the newly formed bone located both in the intercortical and medullary regions. (B) The bone surfaces facing the screw are covered by mature lamellar bone (thin arrows). Osteoid tissues (OS) are seen in deeper regions of the bone located both in the intercortical and medullary regions. Body and threads (thin arrows) of the screw are shown as empty areas (magnification: 100 μm).

At the 6^th^ postoperative week, the defect area of the control group was covered with intact periosteum. The areas between screw threads in both the intercortical and medullary regions were mainly filled with newly formed primary bone tissue (Figure 4). In the experimental group, the head of the screws, located subperiosteally, was surrounded with a thick newly formed bone tissue containing lesser amount of fibrocartilage. The body of the screw located in the intercortical region was surrounded by a large newly formed bone, which was tightly attached to the thread of the screw. Deeper parts of the screws, placed in the bone medullary canal, were surrounded with newly formed lamellar bone. The direction of the newly formed bone lamellae was parallel to the screw axis, whereas perpendicular to the original lamellae. In the experimental group, 25 osteoclastic cells were observed per microscopic field (Table 2). There were no significant differences among the mean percentages of the newly formed bone tissue, osteoid tissue, and connective tissue in the implanted areas in the study groups. Nevertheless, BSA and TbN values of the experimental group were significantly (*p* < 0.05) higher than those of the control group. Blood vessel percentage was significantly (*p* < 0.05) higher than that of the control animals (Table 2). Mean osteoclastic cell count of the control group was 15 cells per microscopic field, which was significantly (*p* < 0.05) lower than that of the experimental group (Table 2).  Graft-versus-host reactions, evidenced by inflammatory changes, encapsulation, or rejection of the coated screws, were not observed in both experimental and control groups (Fig. 4).

## DISCUSSION

In the present study, we observed a porous calcium-phosphate layer on the grade 5 Ti alloy screw surface via the biomimetic coating for a six-hour treatment. Brushite peaks were detected in X-ray diffraction analysis. In addition, the results of the energy dispersive X-ray analysis affirmed the presence of brushite crystals in the coating. After 6 hours of the coating process, the thickness of the coating was compatible with that obtained in previous investigations^[^^18^^,^^19^^]^. It is worth to mention that porous surfaces of hydroxyapatite, calcium-deficient hydroxyapatite, octacalcium phosphate, and brushite (dicalcium phosphate dihydrate) crystals are obtained by the biomimetic coating technique, using a 10× simulated body fluid. Complex geometric materials can also be coated by this method^[^^8^^,^^18^^]^.

Brushite coating layer does not cause a toxic reaction, and a large majority of the crystals are resorbed postoperatively in three weeks^[^^18^^-^^21^^]^. Similarly, in our study, we did not observe the coating layer in the µ-CT sections at the 6^th^ week, postoperatively. Also, there was no fibrous capsulation tissue around the implanted screws. In a study performed by Salama and El-Sakhawy^[^^22^^]^, biodegradable materials such as polylactic acid, carboxymethyl cellulose, and chitosan were used as tissue scaffolds in biomimetic coating, together with calcium phosphate crystals. Their results indicated that these hybrid materials can be used in drug delivery systems^[^^22^^]^. Furthermore, these biodegradable hybrid structures are utilized in tissue engineering applications^[^^22^^,^^23^^]^. 

Using natural or synthetic methods, several hybrid materials were obtained to improve the beneficial properties of various synthetic polymers with osteoconductive capabilities. Therefore, we developed a hybrid surface by cross-linking soluble eggshell membrane proteins onto the brushite coating layer on the grade 5 Ti alloy screw surfaces. Biomimetically, coated calcium phosphate crystals are applied for the controlled release of various bioactive molecules^[^^22^^]^. Therefore, these biomimetic-coated crystals were selected to gradually release soluble eggshell membrane proteins in our in vivo experimental model.

Eggshell membranes contain mainly type I, V, and X collagens, osteopontin, and sialoproteins. However, their solubility is very low due to the presence of cross-linked disulfide bonds, which limit the applications of the product in tissue engineering. Trypsin digestion resulted in a water-soluble material with suitable bioactive feature; hence, it was described as a biocompatible material that promotes collagen matrix formation^[^^10^^]^. Results of previous in vitro studies showed that the water-soluble eggshell membrane proteins are biocompatible and biodegradable bioactive material, which facilitates the adhesion and proliferation of fibroblasts^[^^16^^,^^23^^,^^24^^]^.

In a study, the hydroxyl apatite and gelatin-containing tissue scaffolds was produced by a method carried out by Narbat et al.^[^^25^^]^. In this study, we aimed to examine the in vivo effects of a hybrid surface, which consists of brushite surface coating and cross-linked water-soluble eggshell membrane protein. No signs of toxicity attributable to the coating material and protein were observed in the surrounding tissues. The µ-CT results showed that the mean bone surface density and TbN in the region of interest in the experimental group were significantly higher than those of the control group (*p* ˂ 0.05). Observation of the high bone surface density scores in areas of newly formed boneindicates that the mechanical properties of the newly formed bone in the region have been also strengthened.

**Table 1:** µ-CT results of the control and experimental screws on the 6^th^ week after surgery

**Groups** **(n = 6 each)**	**BV** **(%)**	**BSA** **(mm** ^-1^ **)**	**TbN** **(mm** ^-1^ **)**	**TbSp** **(mm** ^-1^ **)**	**TbTh** **(mm** ^-1^ **)**	**BMD** **(mgHAcm** ^-3^ **)**
Control	12.59 ± 3.5	2.74 ± 0.2	0.67 ± 0.1	1.26 ± 0.1	0.18 ± 0.0	319.5 ± 11.1
Experimental	13.15 ± 1.9	3.57 ± 0.1^*^	0.86 ± 0.1^*^	1.04 ± 0.2	0.15 ±0.0	317.7 ± 16.3

^*^The difference between the values in the same column is statistically significant (*p* < 0.05). Each data represents mean ± SD.

The results of the present study also demonstrated that water-soluble eggshell membrane protein cross-linked to the calcium phosphate coating formed by the biomimetic method, stimulated new bone formation at the implantation site, as evidenced by the significant increase in BSA and TbNs. Moreover, the mean percentages of the newly formed bone, osteoid, and connective tissues of the experimental group were higher than that of the control group, although the differences were statistically nonsignificant. The blood vessel percentages and osteoclast numbers per microscopic area were significantly higher (*p* < 0.05) in the experimental group than the control group. However, due to the relatively high solubility of the calcium phosphate and eggshell membrane protein hybrid in body fluids, the contribution of this hybrid material to new bone formation may be limited. Our findings suggest that brushite crystals and eggshell membrane protein hybrid are rapidly absorbed at the early stages of bone healing in the defect area with high blood flow rates and high metabolic activities. Therefore, it is expected that the beneficial effects will be limited. These results showed that healing process and bone remodeling were at the advanced level in the experimental animals. It is well known that higher blood supply into the damaged tissues accelerates metabolic activities and healing processes in the defect site. Hence, it can be concluded that the increased vascularization accelerates the healing process and the new bone formation rate. Given that we did not observe graft-versus-host reactions, such as inflammatory changes and encapsulation of the coated screws in the experimental group, it is reasonable to consider both the calcium phosphate coating and cross-linked eggshell membrane protein hybrid as biocompatible and bioactive materials.

**Table 2:** Histomorphometrical results of the control and experimental groups on the 6^th^ week of the post operation period

**Groups** **(n = 10 each)**	**Newly formed bone area (%)**	**Osteoid tissue area (%)**	**Connective tissue area (%)**	**Vascular area (%)**	**Osteclastic cell in a single microscope area**
Control	56.09 ± 19.5	28.00 ± 15.7	14.98 ± 10.3	0.92 ± 0.9	15 ± 0.8
Experimental	61.42 ± 16.9	22.05 ± 11.4	12.23 ± 7.7	4.29 ± 2.5^*^	25 ± 0.5^*^

^ *^The difference between the values in the same column was statistically significant. Each data represents mean ± SD.

## CONCLUSION

Our findings reveal that the biomimetically calcium phosphate coating and its cross-linking with water-soluble ostrich eggshell membrane protein enhance the osseointegration of grade 5 Ti alloy screws via increasing new bone formation and vascularization. 

## DECLARATIONS

### Acknowledgments

 The authors thank to the Scientific Research Projects Coordination Unit of Selçuk University, Konya, Türkiye. No artificial intelligence was used in this study. 

### Ethical approval

 All animal care and study protocols were approved by the Institutional Experimental Animal Care and Use Committee of Necmettin Erbakan University, Konya, Türkiye (ethical code: 2014-002/12.02.2014).

### Consent to participate

 Not applicable. 

### Consent for publication

 All authors reviewed the results and approved the final version of the manuscript.

### Authors’ contributions

 MBİ: running the project; ED: advising the project; İÇ: managing the project. 

### Data availability

Data supporting this article are included in the article and are available by the corresponding author on reasonable request.

### Competing interests

The authors declare that they have no competing interests. 

### Funding

The Project was funded by the Scientific Research Projects Coordination Unit of Selçuk University, Konya, Türkiye (grant number: 12202034).

### Supplementary information

The online version does not contain supplementary material.
